# Impact of preoperative mental health disorders on postoperative outcomes in patients with adolescent idiopathic scoliosis undergoing surgery

**DOI:** 10.1007/s43390-026-01288-z

**Published:** 2026-01-26

**Authors:** Emily K. Vallee, Ellen Lutnick, Alexander Yunke, Maxwell M. Scott, Gabrielle A. Orie, Lauren Harte, Allison S. Binkley, Michael R. Ferrick, Jeremy P. Doak

**Affiliations:** 1 Jacobs School of Medicine and Biomedical Sciences, University at Buffalo, 955 Main St, Buffalo, NY 14203 USA; 2 Department of Orthopaedics, Jacobs School of Medicine and Biomedical Sciences, University at Buffalo, Buffalo, NY USA

**Keywords:** Mental health, Complications, Scoliosis, Spinal fusion, Outcomes, Healthcare utilization

## Abstract

**Purpose:**

Mental health disorders (MHD) represent a growing concern in the pediatric population. This study aims to evaluate whether the presence of a mental health disorder influences perioperative and postoperative outcomes in patients with adolescent idiopathic scoliosis (AIS) undergoing surgery.

**Methods:**

Using the TriNetX collaborative network, a retrospective cohort study was conducted including pediatric patients aged 18 and under who underwent surgery for AIS. Patients were divided based on the presence or absence of a preoperative DSM-5 psychiatric diagnosis. 1:1 propensity score matching was applied to balance covariates. Statistical analyses on primary outcomes were performed.

**Results:**

PSM included 913 patients per cohort. MHD patients had higher odds of wound dehiscence at 3 months (OR 1.92; 95% CI 1.08–3.43), 6 months (OR 1.93; 95% CI 1.10–3.39), 1 year (OR 1.85; 95% CI 1.07–3.17), and 2 years (OR 1.74; 95% CI 1.04–2.91). They also had increased risk of implant mechanical breakdown at 1 year (OR 2.65; 95% CI 1.27–5.52) and 2 years (OR 2.41; 95% CI 1.28–4.53). Similarly, they also had higher readmission at 3 months (OR 1.45; 95% CI 1.03–2.04), and ED visits at 2 years (OR 1.31; 95% CI 1.03–1.66). Following Holm–Bonferroni correction, the only outcome that remained significant was mechanical breakdown at 1 year (OR 2.65; 95% CI 1.27–5.52; *p* = 0.049) and 2 years (OR 2.41; 95% CI 1.28–4.53; *p* = 0.035).

**Conclusions:**

Mental health disorders in AIS patients were associated with higher rates of postoperative complications, with only mechanical failure remaining significant after correction. These findings suggest that preoperative mental health disorders may be an important factor in recovery and surgical outcomes. Further research is needed to better understand these associations and to develop targeted perioperative strategies to optimize care for this population.

**Supplementary Information:**

The online version contains supplementary material available at 10.1007/s43390-026-01288-z.

## Introduction

Mental health disorders (MHDs) are increasing among the pediatric population in the United States, representing a growing public health concern [1–3]. Recently, there has been a rising interest in understanding the relationship between mental health diagnoses in this population and surgical outcomes [4–7]. Studies have demonstrated an association between preoperative mental health disorders and increased rates of postoperative complications and in-hospital mortality amongst adult spinal deformity patients [8].

Adolescent idiopathic scoliosis (AIS) is a common spinal deformity, and severe cases often require posterior spinal fusion (PSF) [9, 10]. This invasive procedure is associated with significant postoperative pain and prolonged hospitalization, sometimes necessitating admission to the intensive care unit [11]. Prior literature has identified an association between AIS and MHDs [11–14]. Rates of depression in adolescents with AIS have been reported to exceed the national average, with approximately one in five patients scoring above the Children’s Depression Inventory cutoff score of 13 [15]. Both the spinal deformity itself and the treatment process are believed to contribute to the psychological burden experienced by these patients [12, 16].

Though recent studies have begun to explore the impact of preoperative mental health diagnoses on perioperative and postoperative outcomes in patients with AIS, this area remains largely understudied [12–14]. The primary aim of this study is to evaluate whether the presence of a mental health disorder influences perioperative and postoperative outcomes in patients with AIS undergoing surgical correction, utilizing a large global network database.

## Methods

### Data source and collection

After receiving institutional-review board approval (IRB# STUDY0007312), a retrospective case–control study was performed using the TriNetX Collaborative Network (Cambridge, Massachusetts).

The TriNetX research network is an international collaborative database that aggregates clinical information from over 220 healthcare organizations (HCOs) across 30 countries, representing over 300 million patients [17, 18]. Participating institutions contribute deidentified or limited patient data, which are made available on the TriNetX platform for research use. In return for data participation, HCOs gain access to analytical and visualization tools, along with the necessary technological infrastructure to support these functions. All data shared through TriNetX are deidentified in accordance with the Health Insurance Portability and Accountability Act (HIPAA) Privacy Rule, Sect. 164.514(b)(1), as verified by an expert determination, ensuring full compliance with HIPAA standards. The database contains a wide range of variables, including demographic information, encounter details, diagnostic codes, procedures, and prescribed medications, among others. [17, 18]

### Patient cohort selection and propensity matching

**Fig. 1:**
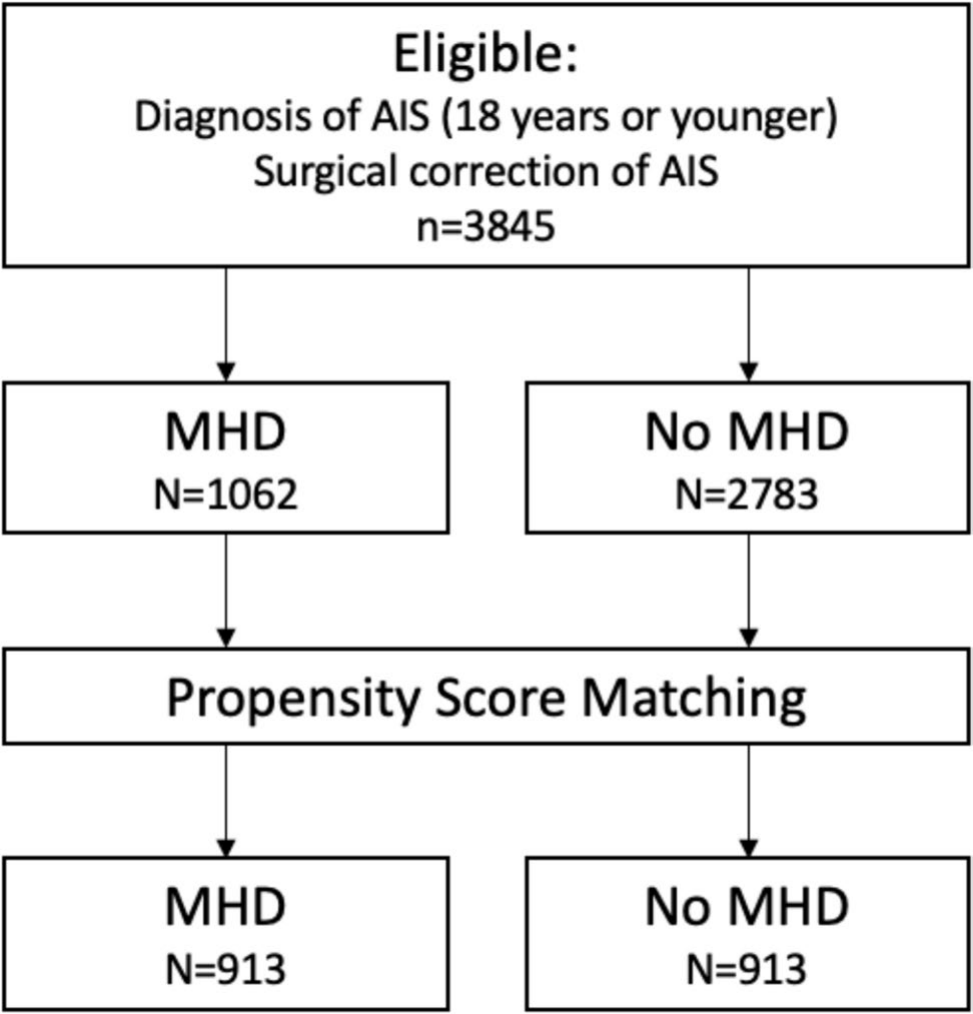
CONSORT flow diagram detailing patient selection and exclusion

On August 15th, 2025, Current Procedure Terminology (CPT) codes 22,800, 22,802, 22,804, 22,808, 22,810, and 22,812 as well as International Classification of Diseases, 10th Edition (ICD-10) code M41.1 for AIS were used to identify patients 18 years or younger who underwent primary surgical correction for AIS between 2005 and August 2025 (Fig. 1). 3,845 AIS patients were discovered for analysis. Patients were separated into case and control groups based on the presence or absence of one or multiple mental or behavior disorder ICD-10 diagnoses: F10–19, F20–F29, F30–F39, F40–F48, F50, F70–F79, and F90–F98. These disease classification codes were used to separate patients with a preoperative MHD from patients with no documented lifetime MHD diagnosis. Specific codes and details pertaining to each query can be found in Supplemental File S1. Following 1:1 propensity matching, 913 patients remained in both cohorts for analysis. 64% (n = 1168/1826) had at least a 1 year follow-up.

### Outcomes

Primary outcomes in this study were various medical and surgical complications (wound dehiscence, surgical site infection, mechanical breakdown, implant infection, postoperative pain, return to the emergency department and to the hospital) occurring at multiple timepoints following the index procedure (1 month, 3 months, 6 months, 1 year, and 2 years). Return to the emergency department (ED) and hospital data were adjusted to start 1 week following the index procedure. Secondary outcomes included sepsis, hemorrhage, respiratory failure, reintubation, paraplegia or quadriplegia, myocardial infarction, iatrogenic stroke, disseminated intravascular coagulation, deep vein thrombosis (DVT), pulmonary embolism, and death. Secondary outcomes were analyzed at 1 month, 3 months, and 6 months timepoints only.

### Data analysis

Continuous variables were compared using two-tailed independent samples *t* tests and expressed as means and standard deviations. Categorical variables were compared using chi-square tests and Fisher’s exact tests where appropriate. They are expressed as numbers and percentages of cohort totals. Nearest propensity score matching was performed at a 1:1 ratio with a caliper of 0.1, matching patients with a preoperative MHD diagnosis to those with no documented lifetime MHD diagnosis. Patients were matched for the demographics, comorbidities, and arthrodesis levels shown in Table 1. Patient characteristics were considered well-matched if the *p* value was less than 0.05. Dichotomous outcomes were assessed using an odds ratio (OR) with a 95% confidence interval (CI). A *p* value less than 0.05 was considered significant. Analysis was performed within the TriNetX collaborative network, with Python (version 3.13) used for Holm–Bonferroni correction following regression analysis.

**Table 1:** Balancing of covariates using propensity score matching

		**Prior to Propensity Matching**				**After Propensity Matching**			
		Patients with History of MHD (n = 1062)	Patients with No History of MHD (n = 2783)	*p* value	Std. diff	Patients with History of MHD (n = 913)	Patients with No History of MHD (n = 913)	*p* value	Std diff
**Demographics**									
Age at surgery (mean ± SD)		13.2 ± 2.4	12.9 ± 2.4	**0.007**	0.096	13.1 ± 2.4	13.2 ± 2.2	0.286	0.050
Sex	Female (n, %)	736 (69.3%)	2111 (75.9%)	** < 0.001**	0.147	649 (71.1%)	645 (70.6%)	0.827	0.010
	Male (n, %)	326 (30.7%)	670 (24.1%)	** < 0.001**	0.149	264 (28.9%)	268 (29.4%)	0.837	0.010
Race	Asian (n, %)	32 (3/0%)	113 (4.1%)	0.127	0.057	29 (3.2%)	24 (2.6%)	0.486	0.033
	American Indian or Alaska Native (n, %)*	10 (0.9%)	10 (0.4%)	**0.025**	0.072	10 (1.1%)	10 (1.1%)	1.000	< 0.001
	Black or African American (n, %)	141 (13.3%)	581 (20.9%)	** < 0.001**	0.149	133 (14.6%)	138 (15.1%)	0.742	0.015
	Native Hawaiian or Other Pacific Islander (n, %)*	10 (0.9%)	10 (0.4%)	**0.025**	0.072	10 (1.1%)	10 (1.1%)	1.000	< 0.001
	Other (n, %)	75 (7.1%)	234 (8.4%)	0.170	0.050	68 (7.4%)	70 (7.7%)	0.859	0.008
	White (n, %)	755 (71.1%)	1658 (59.6%)	** < 0.001**	0.244	632 (69.2%)	626 (68.6%)	0.762	0.014
Ethnicity	Hispanic or Latino (n, %)	94 (8.9%)	312 (11.2%)	**0.033**	0.079	84 (9.2%)	71 (7.8%)	0.275	0.051
	Not Hispanic or Latino (n, %)	843 (79.4%)	2095 (75.3%)	**0.007**	0.098	717 (78.5%)	731 (80.1%)	0.419	0.038
**Comorbidities (n, %)**									
	Diseases of the blood and blood-forming organs and certain disorders involving the immune mechanism (ICD-10: D50–D89)	415 (39.1%)	607 (21.8%)	** < 0.001**	0.382	323 (35.4%)	311 (34.1%)	0.555	0.028
	Congenital malformations, deformations and chromosomal abnormalities (ICD-10: Q00–Q99)	499 (47.0%)	764 (27.5%)	** < 0.001**	0.413	383 (41.9%)	370 (40.5%)	0.537	0.029
	Diseases of the nervous system (ICD-10: G00–G99)	854 (80.4%)	1796 (64.5%)	** < 0.001**	0.361	710 (77.8%)	732 (80.2%)	0.206	0.059
	Diseases of the respiratory system (ICD-10: J00–J99)	706 (66.5%)	1084 (39.0%)	** < 0.001**	0.574	562 (61.6%)	542 (59.4%)	0.338	0.045
	Diseases of the digestive system (ICD-10: K00–K95)	642 (60.4%)	821 (29.5%)	** < 0.001**	0.653	493 (54.0%)	497 (54.4%)	0.851	0.009
	Diseases of the circulatory system (ICD-10: I00–I99)	333 (31.4%)	456 (16.4%)	** < 0.001**	0.357	253 (27.7%)	232 (25.4%)	0.266	0.052
	Diseases of the skin and subcutaneous tissue (ICD-10: L00–L99)	450 (45.5%)	618 (22.2%)	** < 0.001**	0.442	337 (36.9%)	338 (37.0%)	0.961	0.002
	Endocrine, nutritional and metabolic diseases (ICD-10: E00–E99)	483 (45.5%)	656 (23.6%)	** < 0.001**	0.474	367 (40.2%)	351 (38.4%)	0.443	0.036
	Diseases of the genitourinary system (ICD-10: N00–N99)	367 (34.6%)	361 (13.0%)	** < 0.001**	0.524	258 (28.3%)	241 (26.4%)	0.372	0.042
	Other joint disorders (ICD-10: M20–M25)	344 (32.4%)	479 (17.2%)	** < 0.001**	0.357	249 (27.3%)	251 (27.5%)	0.916	0.005
	Systemic connective tissue disorders (ICD-10: M30–M36)	25 (2.4%)	41 (1.5%)	0.060	0.064	21 (2.3%)	20 (2.2%)	0.874	0.007
	Spondylopathies (ICD-10: M45–M49)	40 (3.8%)	61 (2.2%)	**0.006**	0.093	30 (3.3%)	27 (3.0%)	0.686	0.019
	Other dorsopathies (ICD-10: M50–M54)	395 (37.2%)	762 (27.4%)	** < 0.001**	0.211	314 (34.4%)	324 (35.5%)	0.624	0.023
	Disorders of muscles (ICD-10: M60–M63)	282 (26.6%)	308 (11.1%)	** < 0.001**	0.404	197 (21.6%)	191 (20.9%)	0.731	0.016
	Disorders of synovium and tendon (ICD-10: M65–M67)	41 (3.9%)	40 (1.4%)	** < 0.001**	0.151	29 (3.2%)	25 (2.7%)	0.581	0.026
	Other soft tissue disorders (ICD-10: M70–M79)	210 (19.8%)	228 (8.2%)	** < 0.001**	0.339	138 (15.1%)	149 (16.3%)	0.479	0.033
	Disorders of bone density and structure (ICD-10: M80–M85)	95 (8.9%)	69 (2.5%)	** < 0.001**	0.281	50 (5.5%)	51 (5.6%)	0.918	0.005
	Other osteopathies (ICD-10: M86–M90)	103 (9.7%)	119 (4.3%)	** < 0.001**	0.214	67 (7.3%)	75 (8.2%)	0.485	0.033
	Chondropathies (ICD-10: M91–M94)	65 (6.1%)	54 (1.9%)	** < 0.001**	0.214	41 (4.5)	37 (4.1%)	0.643	0.022
	Other disorders of the musculoskeletal system and connective tissue (ICD-10: M95)	50 (4.7%)	73 (2.6%)	**0.001**	0.111	42 (4.6%)	38 (4.2%)	0.647	0.021
**Arthrodesis Levels (n, %)**									
	Posterior, ≤ 6 levels (CPT: 22,800)	137 (12.9%)	359 (12.9%)	1.000	< 0.001	123 (13.5%)	114 (12.5%)	0.531	0.029
	Posterior, 7–12 levels (CPT: 22,802)	568 (53.5)	1705 (61.3%)	** < 0.001**	0.158	512 (56.1%)	533 (58.4%)	0.321	0.047
	Posterior, ≥ 13 levels (CPT: 22,804)	374 (35.2%)	756 (27.2%)	** < 0.001**	0.174	295 (32.2%)	272 (29.8%)	0.266	0.052
	Anterior, 2–3 levels (CPT: 22,808) *	10 (0.9%)	13 (0.5%)	0.088	0.057	10 (1.1%)	10 (1.1%)	1.000	< 0.001
	Anterior, 4–7 levels (CPT: 22,810) *	10 (0.9%)	31 (1.1%)	0.642	0.017	10 (1.1%)	10 (1.1%)	1.000	< 0.001
	Anterior, ≥ 8 levels (CPT: 22,812) *	10 (0.9%)	10 (0.4%)	**0.025**	0.072	10 (1.1%)	10 (1.1%)	1.000	< 0.001

^*^Indicates a variable, where both cases and control cohorts yielded a value of 10 patients or less. TriNetX censors the actual value of patients with this outcome to maintain HIPAA compliance. Comparative analyses were performed in this case

*Bolded* values indicate statistical significance

## Results

### Demographics

A total of 3,845 who underwent surgical correction for AIS were included, with 1,062 (27.6%) having a documented preoperative MHD diagnosis (Table 2). Prior to matching, patients with a preoperative MHD were significantly older (13.2 ± 2.4 vs. 12.9 ± 2.4; *p* < 0.007), more likely to be male (30.7% vs. 24.1%; *p* < 0.001), and more likely to be White (71.1% vs. 59.6%; *p* < 0.001). They also were less likely to under 7 to 12 levels PSF (53.5% vs. 61.3%; *p* < 0.001), more likely to undergo greater than or equal to 13 levels PSF (35.2% vs. 27.2%; *p* < 0.001), and more likely to undergo greater than or equal to 8 levels anterior fusion (0.9% vs. 0.4%; *p* = 0.025). They also differed significantly in all comorbidities except for systemic connective tissue disorders (2.4% vs. 1.5%; *p* = 0.06). No variables remained significant following propensity matching (Table 1).

**Table 2:** Proportion of mental health diagnoses prior to propensity matching

ICD-10 Code	Diagnosis	*N* = 1062
F10-F19	Mental and behavioral disorders due to psychoactive substance use	3.2% (34)
F20-F29	Schizophrenia, schizotypal, delusional, and other non-mood psychotic disorders	0.8% (9)
F30-F39	Mood (affective) disorders	18.4% (195)
F40-F48	Anxiety, dissociative, stress-related, somatoform and other non-psychotic mental disorders	64.4% (684)
F50	Eating disorders	4.1% (44)
F70-79	Intellectual disabilities	13.3% (141)
F90-98	Behavioral and emotional disorders with onset usually occurring in childhood and adolescence	51.2% (544)

### Complications

Patients with a prior history of MHD had higher odds of wound dehiscence at 3 months (OR 1.923; 95% CI 1.078–3.431; *p* = 0.024), 6 months (OR 1.931; 95% CI 1.099–3.393; *p* = 0.02), 1 year (OR 1.845; 95% CI 1.074–3.169); *p* = 0.024), and 2 years (OR 1.742; 95% CI 1.043–2.907; *p* = 0.032) postoperatively. They also had higher odds of experiencing implant mechanical breakdown at 1 year (OR 2.647; 95% CI 1.269–5.521; *p* = 0.007) and 2 years (OR 2.408; 95% CI 1.280–4.531, *p* = 0.005) postoperatively. Similarly, the same patients had higher odds of returning to the hospital at 3 months (OR 1.452; 95% CI 1.032–2.044; *p* = 0.032) and returning to the ED at 2 years (OR 1.305; 95% CI 1.027–1.657; *p* = 0.029) postoperatively (Table 3).

**Table 3:** Propensity matched postoperative outcomes

1 month								
Outcome (*n*, %)	Patients with History of MHD (Cases) (*n* = 913)	% Risk in Cases	Patients with No History of MHD (Controls) (*n* = 913)	`% Risk in Controls	OR	95%CI	Unadjusted *p* value	Adjusted *p* value
Wound Dehiscence*	19	2.1%	10	1.1%	1.919	(0.887, 4.150)	0.092	1.000
Surgical Site Infection	11	1.2%	12	1.3%	0.916	(0.402, 2.086)	0.834	1.000
Mechanical Breakdown*	14	1.5%	10	1.1%	1.406	(0.621, 3.182)	0.411	1.000
Implant Infection*	10	1.1%	10	1.1%	1.000	(0.414, 2.414)	1.000	1.000
Postoperative Pain	433	47.4%	474	51.9%	0.835	(0.695, 1.004)	0.055	0.660
Thrombosis and Embolism	0	0.0%	0	0.0%	-	-	-	-
Sepsis*	10	1.1%	10	1.1%	1.000	(0.414, 2.414)	1.000	1.000
Hemorrhage*	10	1.1%	10	1.1%	1.000	(0.414, 2.414)	1.000	1.000
Respiratory Failure	45	4.9%	47	5.1%	0.955	(0.628, 1.453)	0.831	1.000
Death‡	10	1.1%	0	0.0%	-	-	-	-
Reintubation*	10	1.1%	10	1.1%	1.000	(0.414, 2.414)	1.000	1.000
Pulmonary Embolism‡	0	0.0%	10	1.1%	-	-	-	-
Paraplegia and Quadriplegia*	10	1.1%	15	1.6%	0.663	(0.296, 1.484)	0.314	1.000
Myocardial Infarction	0	0.0%	0	0.0%	-	-	-	-
Iatrogenic Stroke‡	10	1.1%	0	0.0%	-	-	-	-
Deep Vein Thrombosis*	10	1.1%	10	1.1%	1.000	(0.414, 2.414)	1.000	1.000
Shock*	10	1.1%	10	1.1%	1.000	(0.414, 2.414)	1.000	1.000
Disseminated Intravascular Coagulation*	10	1.1%	10	1.1%	1.000	(0.414, 2.414)	1.000	1.000
Return to the Emergency Department	53	5.8%	38	4.2%	1.419	(0.926, 2.175)	0.107	1.000
Return to the Hospital	53	5.8%	46	5.0%	1.162	(0.774, 1.743)	0.469	1.000

**3 months**								
Outcome (*n*, %)	Patients with History of MHD (Cases) (*n* = 913)	% Risk in Cases	Patients with No History of MHD (Controls) (*n* = 913)	% Risk in Controls	OR	95%CI	Unadjusted *p* value	Adjusted *p* value
Wound Dehiscence*	34	3.7%	18	2.0%	1.923	(1.078, 3.431)	**0.024**	0.360
Surgical Site Infection	20	2.2%	16	1.8%	1.256	(0.646, 2.439)	0.501	1.000
Mechanical Breakdown*	18	2.0%	10	1.1%	1.816	(0.834, 3.956)	0.128	1.000
Implant Infection*	12	1.3%	13	1.4%	0.922	(0.418, 2.032)	0.840	1.000
Postoperative Pain	435	47.6%	475	52.0%	0.839	(0.698, 1.008)	0.061	0.793
Thrombosis and Embolism	0	0.0%	0	0.0%	-	-	-	-
Sepsis*	10	1.1%	10	1.1%	1.000	(0.414, 2.414)	1.000	1.000
Hemorrhage*	10	1.1%	10	1.1%	1.000	(0.414, 2.414)	1.000	1.000
Respiratory Failure	51	5.6%	51	5.6%	1.000	(0.671, 1.491)	1.000	1.000
Death‡	10	1.1%	0	0.0%	-	-	-	-
Reintubation*	10	1.1%	10	1.1%	1.000	(0.414, 2.414)	1.000	1.000
Pulmonary Embolism‡	0	0.0%	10	1.1%	-	-	-	-
Paraplegia and Quadriplegia*	19	2.1%	19	2.1%	1.000	(0.526, 1.901)	1.000	1.000
Myocardial Infarction	0	0.0%	0	0.0%	-	-	-	-
Iatrogenic Stroke‡	10	1.1%	0	0.0%	-	-	-	-
Deep Vein Thrombosis*	10	1.1%	10	1.1%	1.000	(0.414, 2.414)	1.000	1.000
Shock*	10	1.1%	12	1.3%	0.831	(0.357, 1.934)	0.668	1.000
Disseminated Intravascular Coagulation*	10	1.1%	10	1.1%	1.000	(0.414, 2.414)	1.000	1.000
Return to the Emergency Department	53	5.8%	38	4.2%	1.132	(0.817, 1.568)	0.455	1.000
Return to the Hospital	86	9.4%	61	6.7%	1.452	(1.032, 2.044)	**0.032**	0.448

6 months								
Outcome (*n*, %)	Patients with History of MHD (Cases) (*n* = 913)	% Risk in Cases	Patients with No History of MHD (Controls) (*n* = 913)	% Risk in Controls	OR	95%CI	Unadjusted *p* value	Adjusted *p* value
Wound Dehiscence*	36	3.9%	19	2.1%	1.931	(1.099, 3.393)	**0.020**	0.300
Surgical Site Infection	23	2.5%	17	1.9%	1.362	(0.723, 2.567)	0.337	1.000
Mechanical Breakdown*	20	2.2%	10	1.1%	2.022	(0.941, 4.345)	0.066	0.924
Implant Infection*	12	1.3%	13	1.4%	0.922	(0.418, 2.032)	0.840	1.000
Postoperative Pain	440	48.2%	479	52.5%	0.843	(0.701, 1.013)	0.068	0.924
Thrombosis and Embolism	0	0.0%	0	0.0%	-	-	-	-
Sepsis*	10	1.1%	11	1.2%	0.908	(0.384, 2.149)	0.826	1.000
Hemorrhage*	10	1.1%	10	1.1%	1.000	(0.414, 2.414)	1.000	1.000
Respiratory Failure	53	5.8%	52	5.7%	1.020	(0.688, 1.513)	0.920	1.000
Death‡	10	1.1%	10	1.1%	1.000	(0.414, 2.414)	1.000	1.000
Reintubation*	10	1.1%	10	1.1%	1.000	(0.414, 2.414)	1.000	1.000
Pulmonary Embolism‡	0	0.0%	10	1.1%	-	-	-	-
Paraplegia and Quadriplegia*	20	2.2%	22	2.4%	0.907	(0.492, 1.674)	0.755	1.000
Myocardial Infarction	0	0.0%	0	0.0%	-	-	-	-
Iatrogenic Stroke‡	10	1.1%	0	0.0%	-	-	-	-
Deep Vein Thrombosis*	10	1.1%	10	1.1%	1.000	(0.414, 2.414)	1.000	1.000
Shock*	10	1.1%	12	1.3%	0.831	(0.357, 1.934)	0.668	1.000
Disseminated Intravascular Coagulation*	10	1.1%	10	1.1%	1.000	(0.414, 2.414)	1.000	1.000
Return to the Emergency Department	99	10.8%	82	9.0%	1.233	(0.906, 1.677)	0.183	1.000
Return to the Hospital	97	10.6%	93	10.2%	1.048	(0.776, 1.416)	0.759	1.000

1 year								
Outcome (*n*, %)	Patients with History of MHD (Cases) (*n* = 913)	% Risk in Cases	Patients with No History of MHD (Controls) (*n* = 913)	% Risk in Controls	OR	95%CI	Unadjusted *p* value	Adjusted *p* value
Wound Dehiscence	38	4.2%	21	2.3%	1.845	(1.074, 3.169)	**0.024**	0.144
Surgical Site Infection	25	2.7%	20	2.2%	1.257	(0.693, 2.280)	0.450	0.900
Mechanical Breakdown	26	2.8%	10	1.1%	2.647	(1.269, 5.521)	**0.007**	**0.049**
Implant Infection	14	1.5%	14	1.5%	1.000	(0.474, 2.110)	1.000	1.000
Postoperative Pain	449	49.2%	485	53.1%	0.854	(0.711, 1.026)	0.092	0.390
Return to the Emergency Department	139	15.2%	113	12.4%	1.271	(0.973, 1.661)	0.078	0.390
Return to the Hospital	123	13.5%	108	11.8%	1.161	(0.880, 1.530)	0.291	0.873

2 years								
Outcome (*n*, %)	Patients with History of MHD (Cases) (*n* = 913)	% Risk in Cases	Patients with No History of MHD (Controls) (*n* = 913)	% Risk in Controls	OR	95%CI	Unadjusted *p* value	Adjusted *p* value
Wound Dehiscence	41	4.5%	24	2.6%	1.742	(1.043, 2.907)	**0.032**	0.174
Surgical Site Infection	28	3.1%	21	2.3%	1.344	(0.757, 2.384)	0.311	0.622
Mechanical Breakdown	33	3.6%	14	1.5%	2.408	(1.280, 4.531)	**0.005**	**0.035**
Implant Infection	16	1.8%	17	1.9%	0.940	(0.472, 1.872)	0.861	0.861
Postoperative Pain	454	49.7%	494	54.1%	0.839	(0.698, 1.008)	0.061	0.244
Return to the Emergency Department	184	20.2%	148	16.2%	1.305	(1.027, 1.657)	**0.029**	0.174
Return to the Hospital	155	17.0%	132	14.5%	1.210	(0.940, 1.558)	0.139	0.417

^*^Indicates an outcome, where both cases and control cohorts yielded a value of 10 patients. TriNetX censors the actual value of patients with this outcome to maintain HIPAA compliance. Regression analysis was performed in this case, all of these results are insignificant because of this

^‡^ Indicates an outcome, where one of the case or control cohorts yielded a value of 10 patients. Regression analyses were not performed in this case

*Bolded* values indicate statistical significance

No statistically significant association was observed between MHD status and postoperative pain at 1 month (OR 0.835; 95% CI 0.695–1.004; *p* = 0.055), 3 months (OR 0.839; 95% CI 0.698–1.008; *p* = 0.061), 6 months (OR 0.843; 95% CI 0.701–1.013; *p* = 0.068), 1 year (OR 0.854, 95% CI 0.711–1.026; *p* = 0.092), and 2 years (OR 0.839; 95% CI 0.698–1.008; *p* = 0.061) postoperatively (Table 3).

### Post-hoc analysis

After performing the Holm–Bonferroni correction, the only outcome that remained statistically significant was mechanical breakdown at 1 year between compared groups (OR 2.65; 95% CI 1.27–5.52; *p* = 0.049) and 2 years (OR 2.41; 95% CI 1.28–4.53; *p* = 0.035) postoperatively.

## Discussion

The impact of MHDs on surgical outcomes among the pediatric population is an area of increased interest and concern amongst medical research. However, the relationship between MHDs and AIS is not well-understood. Given that surgical correction of AIS is invasive with potential for serious complications following surgery, the perioperative and postoperative outcomes in patients with known MHD diagnoses prior to surgical correction were examined. In this propensity matched analysis, patients were identified with a preoperative MHD experienced significantly higher odds of various surgical complications including wound dehiscence, implant mechanical breakdown, and increased healthcare utilization following surgical correction of AIS, with mechanical failure remaining significant after Holm–Bonferroni correction.

These findings are comparable to prior literature examining the relationship between psychiatric comorbidities and postoperative outcomes. In adult patients, MHDs have functioned as independent predictors of increased postoperative morbidity, prolonged hospitalization, and increased complication rates [8, 19]. In pediatric patients, similar trends have been reported recently in the literature. Anastasio et al. reported an increased odds ratio for increase length of stay for AIS patients with either depression or anxiety [13]. Similarly, Abu-Zahra et al. reported greater healthcare utilization among AIS patients with MHDs, though complication rates were comparable between those with and without a preoperative MHD diagnosis [14]. Earlier single-center studies that did not identify these associations were likely limited by small sample sizes, short follow-up durations, or inadequate covariate adjustment [20, 21]. Using a large international database with extended follow-up and robust propensity matching, this analysis suggests that psychiatric comorbidity is an independent risk factor for various surgical complications and increased healthcare utilization after surgical correction for AIS.

The mechanisms underlying the relationship between MHD and poorer outcomes among AIS patients undergoing surgical correction is likely multifactorial. From a behavior standpoint, patients with MHDs may be less compliant with postoperative activity limitations, medication regimens, or wound care, which can place added stress on the surgical construct and healing wound, thereby elevating the risk of mechanical or wound-related complications [19, 22–24]. Physiologically, research suggests chronic psychological stress and affective disorders can disrupt the hypothalamic–pituitary–adrenal axis and elevate systemic inflammatory cytokines, leading to impaired immune function and delayed tissue repair [25–28]. These alterations may hinder wound healing and fusion integrity, contributing to the higher rates of wound dehiscence and implant mechanical breakdown seen in this study’s MHD population.

This analysis did not demonstrate statistically significant differences in postoperative pain between cohorts, which contrasts with previous literature. Connelly et al. found anxiety to be a predictor of slower improvement in pain following surgical correction of AIS, while Bennett et al. reported that AIS patients undergoing surgery with a preoperative MHD experienced higher average pain scores and patient-controlled analgesia demands [11, 20]. The absence of an association in the present study likely reflects a limitation of the TriNetX database, which does not capture patient-reported outcomes, detailed analgesic use, or other complex biopsychosocial factors. In addition, postoperative opioid and nonsteroidal anti-inflammatory drug use were excluded from our analysis to minimize bias from institutional prescribing variability and incomplete data capture, which may have obscured subtle but clinically relevant differences. Multiple studies, including that by Abu-Zahra et al., have demonstrated higher postoperative pain scores, greater analgesic use, and prolonged opioid dependence among adolescents with various MHDs, including depression and anxiety [14, 20, 29]. The relationship between MHDs and increased postoperative pain following surgical correction of AIS is, therefore, likely to be clinically significant, despite the lack of statistical significance observed in our findings.

This study has several limitations. First, the TriNetX database relies on administrative coding and data extracted from electronic health records across multiple institutions, introducing the potential for coding errors and under-reporting of complications. ICD codes may also lack sensitivity to capture all relevant adverse events, as well as lacking the sensitivity to capture the specific phases of mental health disorders at the time of operative management with regard to level of MHD or severity, or regarding response to treatment, which may have caused results to vary. Given the nature of the source of the data included for analysis, there is no way ensure under-reporting or misdiagnoses, and no way to determine the level of training of the healthcare providers who diagnosed the MHDs captured. Unplanned return to the operating room was also not a data point available for analysis.

Despite these limitations, we observed significantly higher odds of perioperative and postoperative complications among patients with mental health disorders undergoing surgical correction for AIS. Second, patient-reported outcomes and subjective measures such as postoperative pain were unavailable, limiting assessment of these important recovery metrics. The database also lacks specific diagnostic information regarding hospital readmissions or emergency department visits, which may influence interpretation of the results. Future studies should incorporate detailed readmission data and patient-reported outcomes to better characterize this population and inform strategies to optimize perioperative care.

## Conclusion

This study found patients undergoing surgical correction for AIS with a MHD diagnosis had higher odds of having wound dehiscence, implant mechanical breakdown, readmission to the hospital, and return to the emergency department at various timepoint after surgical correction for AIS. These findings suggest that MHDs may significantly influence postoperative recovery and surgical outcomes. The association between MHDs and adverse events may be related to factors, such as altered pain perception, reduced treatment adherence, or physiologic stress responses that negatively impact wound healing and rehabilitation. Further investigation is needed to better define these relationships and to develop targeted, multidisciplinary strategies aimed at improving both mental health and surgical outcomes in this high-risk population.

## Supplementary Information

Below is the link to the electronic supplementary material.

Supplementary file1 (DOCX 16 KB)

## Data Availability

The dataset for this study is available from the corresponding author (Vallee EK) upon reasonable request.
