# Supplementary material for: Impact of preoperative mental health disorders on postoperative outcomes in patients with adolescent idiopathic scoliosis undergoing surgery
Source: Spine Deform. 2026 Jan 26;14(4):1319–30. doi: 10.1007/s43390-026-01288-z (PMC13323283; doi:10.1007/s43390-026-01288-z)
Supplement: Supplementary file 1 — Supplementary file1 (DOCX 16 KB) [file 43390_2026_1288_MOESM1_ESM.docx]

**SUPPLEMENTAL FILES:**

| **S1:** Specific Codes for Queries | |
| --- | --- |
| **Outcome** | **ICD-10, CPT, and TriNetX Code(s)** |
| Wound Dehiscence | T81.30, T81.31, T81.32 |
| Surgical Site Infection | T81.40, T81.41, T81.42, T81.49 |
| Mechanical Breakdown | T84.2, T84.3, T84.4 |
| Implant Infection | T84.7, T84.63 |
| Postoperative Pain | G89.18, T84.84 |
| Thrombosis and Embolism | T84.81, T84.86 |
| Sepsis | A41, T81.44 |
| Hemorrhage | R58, T84.83 |
| Respiratory Failure | J96, J95.82 |
| Death | "Deceased" in Demographics |
| Reintubation | 31500 |
| Pulmonary Embolism | I26 |
| Paraplegia and Quadriplegia | G82 |
| Myocardial Infarction | I21, I22 |
| Iatrogenic Stroke | I97.81, I97.82, I63 |
| Deep Vein Thrombosis | I82.4 |
| Shock | T81.1 |
| Disseminated Intravascular Coagulation | D65 |
| Return to the Emergency Department | 1013711, "Emergency" in Visits |
| Return to the Hospital | 1013675, 1013699, "Inpatient Encounter" in Visits, "Inpatient Acute" in Visits |
